# Quality control and quality assessment of data from surface-enhanced laser desorption/ionization (SELDI) time-of flight (TOF) mass spectrometry (MS)

**DOI:** 10.1186/1471-2105-6-S2-S5

**Published:** 2005-07-15

**Authors:** Huixiao Hong, Yvonne Dragan, Joshua Epstein, Candee Teitel, Bangzheng Chen, Qian Xie, Hong Fang, Leming Shi, Roger Perkins, Weida Tong

**Affiliations:** 1 Division of Bioinformatics, Z-Tech at FDA's National Center for Toxicological Research, Jefferson, Arkansas 72079, USA; 2 Division of Systems Toxicology, FDA's National Center for Toxicological Research, Jefferson, Arkansas 72079, USA; 3 Myleoma Institute for Research and Therapy, University of Arkansas for Medical Sciences, Arkansas Cancer Research Center, Little Rock, Arkansas 72205, USA; 4 Division of Molecular Epidemiology, FDA's National Center for Toxicological Research, Jefferson, Arkansas 72079, USA

## Abstract

**Background:**

Proteomic profiling of complex biological mixtures by the ProteinChip technology of surface-enhanced laser desorption/ionization time-of-flight (SELDI-TOF) mass spectrometry (MS) is one of the most promising approaches in toxicological, biological, and clinic research. The reliable identification of protein expression patterns and associated protein biomarkers that differentiate disease from health or that distinguish different stages of a disease depends on developing methods for assessing the quality of SELDI-TOF mass spectra. The use of SELDI data for biomarker identification requires application of rigorous procedures to detect and discard low quality spectra prior to data analysis.

**Results:**

The systematic variability from plates, chips, and spot positions in SELDI experiments was evaluated using biological and technical replicates. Systematic biases on plates, chips, and spots were not found. The reproducibility of SELDI experiments was demonstrated by examining the resulting low coefficient of variances of five peaks presented in all 144 spectra from quality control samples that were loaded randomly on different spots in the chips of six bioprocessor plates. We developed a method to detect and discard low quality spectra prior to proteomic profiling data analysis, which uses a correlation matrix to measure the similarities among SELDI mass spectra obtained from similar biological samples. Application of the correlation matrix to our SELDI data for liver cancer and liver toxicity study and myeloma-associated lytic bone disease study confirmed this approach as an efficient and reliable method for detecting low quality spectra.

**Conclusion:**

This report provides evidence that systematic variability between plates, chips, and spots on which the samples were assayed using SELDI based proteomic procedures did not exist. The reproducibility of experiments in our studies was demonstrated to be acceptable and the profiling data for subsequent data analysis are reliable. Correlation matrix was developed as a quality control tool to detect and discard low quality spectra prior to data analysis. It proved to be a reliable method to measure the similarities among SELDI mass spectra and can be used for quality control to decrease noise in proteomic profiling data prior to data analysis.

## Background

Recent advances in proteomic profiling technologies, such as SELDI-TOF MS (Ciphergen Biosystems, Inc., Fremont, CA, ), have allowed preliminary profiling and identification of biomarkers in biological fluids for biological, toxicological, and clinical research [1-10]. ProteinChip technology coupled with SELDI-TOF MS is an effective tool for the simultaneous detection of the relative expression levels of proteins over a wide range of molecular weights in biological samples under different conditions. Differences in protein expression level can then be used to identify disease, differentiate different stages of a disease, toxicant treatment versus control, or different time points following toxicant treatment [11-14].

Analysis of SELDI-TOF MS data presents challenges similar to those for gene expression profile analysis from microarray technologies. Global profiling analyses strive to identify reliable and reproducible expression patterns that are signatures specific to each state, such as disease versus healthy control or different experimental conditions (e.g. treated with a toxicant of interest versus untreated). The identification of biomarkers for diagnosis or prognosis is dependent on analysis of the highly dimensional protein expression profiles. Data must be correctly analyzed before valid interpretation and reliable biological conclusions to be drawn from a protein expression profiles. Analysis of poor quality, noise laden protein expression profiles, however, will likely lead to results lacking biological relevance. Therefore, quality assessment of the protein expression profiles and determination of reproducibility of SELDI-TOF MS experiments and profiles prior to data analysis is of critical importance.

Using SELDI-TOF MS coupled with protein chip technologies for biomarker development is a complicated process that involves many steps, including sample collection and preparation, protein chip selection and preparation, matrix selection and application, spectral calibration, loading sample on chip, washing away non-specifically bound proteins, SELDI-TOF MS parameter settings, data recording, and data pre-processing. Any of these many steps could introduce noise, thus, adversely affecting the quality of the experiment and the reliability of the protein expression profile. A high degree of variability of protein expression profiles in SELDI experiments is not infrequent. The coefficient of variation for absolute intensity measures can be as high as 50–60% [15]. Scientists have recently realized that quality control (QC) is an important issue in SELDI experiments and several efforts have been made to apply some QC techniques to improve the reproducibility of SELDI profiling data [3,16,17]. For example, QC samples that are pooled from multiple samples have been used to assess the reproducibility of a SELDI experiment [18-20], while technical replicates have been used to assess the reproducibility within the same samples [21]. Because of the complex nature of SELDI-TOF MS – ProteinChip experiments, even with experimental QC, the resultant data must be subjected to stringent quality assessment prior to data analysis. Specifically, low quality spectra should be identified and eliminated from analysis to ensure the reliability of biomarkers and the associated patterns discovered during analysis. For example, systematic variability in experiments may introduce additional error sources into the data and this possibility should be examined prior to data analysis.

We investigated systematic variability for plates, chips, and spot positions in two independent SELDI biomarker studies. All peaks (five in our study) appearing in all QC samples should be used to assess the reproducibility of experiments as recommended by Ciphergen because those peaks are the common proteins for the QC samples and should be in similar levels of expression. The high level of reproducibility of our experiments was demonstrated by low coefficients of variation for the five peaks that appeared in all 144 spectra from QC samples. No systematic bias in the experiments was detected (there is no single source of variation that is consistent when switching samples between spots, plates and chips). To identify spectra of low quality, a Pearson correlation matrix was developed as a QC tool to detect low quality spectra in SELDI profiling data analysis, using all peaks in all spectra. The rationale behind the use of a correlation matrix as a QC tool is the assumption that protein expression profiles from biological replicates and technical replicates should be similar. Thus, the correlation matrix is a measure of the similarities among the spectra and useful for quantifying how consistently the experiments have been conducted. We applied the correlation matrix to the SELDI data from the study of biomarkers for liver cancer and liver toxicity, as well as myeloma-associated lytic bone disease. We found that the correlation matrix was an efficient and reliable means to detect low quality spectra should be removed. Doing so should result in more reliable biomarker identification in the final protein expression profiles.

## Methods

### Samples

#### Liver cancer and liver toxicity study

Quality control samples. Plasma was collected in Li-Heparin tubes from two individuals (one male, one female, one Caucasian, one Oriental). Following isolation of plasma from whole blood, all tubes were pooled, mixed, aliquoted into eppendorf tubes, and stored frozen at -80°C. Each tube contained between 80–100 μl of plasma. Four tubes were used for each 96 well plate, one spot per chip. Once thawed, the remaining sample was discarded.

#### Myeloma-associated lytic bone disease

Sera from 64 newly diagnosed myeloma patients were collected during routine clinic visit (Institutional Review Board approved and signed informed consents are kept on record). 36 samples were from patients with 1 to 26 lytic bone lesions, determined by X-ray skeletal surveys, and 28 from patients with no evidence of lytic bone disease

### SELDI protein profiling

Liver cancer and liver toxicity study: The plasma samples were analyzed by SELDI-TOF MS on a Ciphergen ProteinChip Biology System II (PBS II). Specifically, 10 μl of unfractionated samples were used in duplicate for the nonfractionated analyses and 20 μl was used for the fractionation step. Unfractionated and fractionated samples were examined on a weak cation exchange chip (CM10). Two solubilization steps were used for each of the samples whether or not fractionation was to be performed: 9 M urea, 2% CHAPS, 50 μM Tris, pH 9; and 1 M urea, 0.22% CHAPS, 50 μM Tris, pH9. In addition, each of the plasma samples was fractionated for analysis. The plasma to be fractionated was subjected to anion exchange chromatography with stepwise pH elution into 6 fractions [23], each of these fractions was analyzed on a CM10 chip. Plasma (20 μl) was centrifuged for 10 minutes and mixed with the chaotropic urea/CHAPS denaturing and solubilization buffer (30 μl). Each sample was run in duplicate. Sinapinic acid (SPA) was used as the matrix for each sample. The chips were read at two different laser energies to permit ionization and visualization of proteins.

Myeloma-associated lytic bone disease: The serum samples were analyzed by SELDI-TOF MS on a Ciphergen PBS II, using IMAC30 ProteinChips (Ciphergen Biosystems, Inc.) activated with CuSO_4 _according to Ciphergen's protocols. All sera were assayed in quadruplicate and positioned on the 96 spots on each of the 12 chips in a bioprocessor to allow for variability between spots to be determined. For consistency, a BioMek 2000 liquid-handling robot (Beckman-Coulter, Fullerton, CA) was used. SPA was the energy-absorbing matrix. Proteins above 1000 Kda m/z were interrogated using an average of 66 laser shots with laser intensity of 195 and sensitivity of 8. Low molecular weight (MW) standards were assayed with each run (12 chips/run). Calibrations were done for each run.

### Spectral processing

The raw SELDI mass spectra were pre-processed prior to subsequent analysis of the expression profiles. Ciphergen Express 2.0 software was used to calibrate all the spectra in the myeloma-associated lytic bone disease study. Normalization by Total Ion Current was applied to all spectra in both the liver cancer and liver toxicity study and the myeloma-associated lytic bone disease study to minimize the variability in spectra obtained from different times using Ciphergen ProteinChip 3.2 software. The normalization process took the Total Ion Current used for all the spots, averaged the intensity, and adjusted the intensity scales for all the spots so that all spectra could be displayed on the same scale. Baseline subtraction was conducted prior to normalization, as recommended by Ciphergen. Baseline subtraction offsets in the spectra were the result of both electrical noise and the noise from Energy Absorbing Molecules (EAM). The lowest spectral amplitude was detected and then was used to correct the peak height and area. The Biomarker Wizard function in Ciphergen ProteinChip 3.2 software was used to autodetect the peaks present in all of the spectra. The spectral region from 0 to 2500 Da is unreliable for both normalization and peak detection due to matrix interference and was therefore not included in the analysis. A signal to noise ratio greater than 5 was used for the first pass selection of peaks and signal to noise ratio greater than 3 was used for the second pass. Cluster mass window of 0.3% was used for both studies. A requirement to be in a minimum of 100% of samples was set for clusters generation for quality control samples used in liver cancer and liver toxicity study because the common proteins were expected to be expressed in all of the samples. In myeloma-associated lytic bone disease study the requirement was set to be in a minimum of 5% samples for clusters generation because different patient samples were assayed and different protein expressions for different patients were expected.

### Data analysis

After spectral pre-processing using ProteinChip 3.2 software, data was exported as a profile matrix with rows representing spectra and columns representing peak intensities. Principal component analysis (PCA) was performed using Spotfire DecisionSite 7.1 software (Somerville, MA, ). Pearson correlation matrix and other statistical calculations were performed with JMP 5.1 software . Heat maps and correlation matrix surfaces were created with DMVS 2.0 software (Shenzhen Chipscreen Biosciences, Ltd, Shenzhen, P. R. China, ).

## Results

### Assessment of systematic variability from plates, chips, and spots

**Figure 1:**
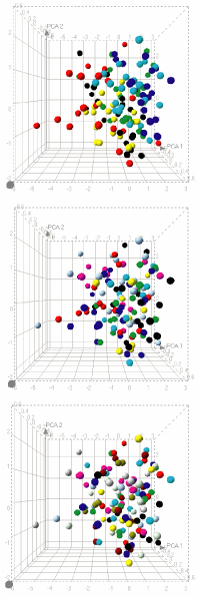
**Assessment of systematic variability across plates, chips, and spots using principal component analysis (PCA) on the 144 quality control samples used in the liver cancer and liver toxicity study. **The 144 spectra obtained from assays of quality control samples from the liver cancer and liver toxicity study were first normalized. The five peaks which appeared in all 144 spectra were taken as the common proteins and used for PCA analysis using software Spotfire DecisionSite 7.1 (Somerville, MA, ). (**A**) The PCA results were color coded for the six Ciphergen bioprocessor plates used to assay the samples. The points represent the spectra and are spread randomly throughout the space. The spectra from each plate did not group together indicating a lack of systematic variability. (**B**) The PCA results were color coded for spot position. Each protein chip has eight spots. The spectra obtained from samples loaded on any same spot position can not be grouped together and randomly spread around in the PCA figure. Thus, spot position did not introduce systematic variability in the data. (**C**) The PCA results were color coded for the chip number in which each sample was loaded for SELDI analysis. Total 12 chips were used for one bioprocessor plate. The spectra of samples from any chips were not grouped together in the PCA, but rather were spread randomly in the space, indicating a lack of systematic variability due to the chip used to assay samples.

SELDI-TOF MS is widely used to profile protein expression in clinical samples including from diseased versus healthy individuals. Analysis of the profiles aims to extract protein biomarkers from the associated patterns that differentiate one group from another. To ensure that the variation in spectra reflects differences between samples rather than systematic variability, assessment of the variability across different plates, chips, and spot positions in protein chips is necessary and important. In our SELDI studies of liver cancer, liver toxicity and lytic bone disease, plasma and sera from patients and controls were applied to the 96 spots on 12 chips on each of the six bioprocessor plates, and the chips read on the PBSII ProteinChip reader (whole data set not shown here). In order to evaluate the systematic variability between plates, chips, and spots, as well as to assess the experiment quality and reproducibility, QC samples were used. Specifically, multiple plasma samples were pooled and used as biological replicates. 144 QC samples were randomly placed on spots of the 12 chips used for each of the six bioprocessor plates. SELDI-TOF MS was then applied to the samples to record the mass spectra. Spectra were first normalized using total ion current (total protein read), and then peaks within a molecular weight range of 2,000 to 40,000 Da were detected and extracted. The peaks present in the spectra of all 144 samples were then used for further analysis. These peaks were expected to be the common proteins or peptides in the QC samples. Principal component analysis (PCA) [22], which is widely employed in signal processing, statistics and neural computing to examine the maximum variability in a highly dimensional data, was used to investigate the source of variance. The PCA results are presented in Fig. 1.

Fig. 1A shows PCA results for data points representing spectra of the 144 QC samples with color denoting bioprocessor plate. The dispersion of the points and lack of clustering indicates that variability across plates is random. Fig. 1B similarly shows the PCA results for all 144 QC samples with color denoting one of the eight different spot positions. Again, dispersion and lack of grouping indicates the absence of systematic variability across position. Fig. 1C similarly shows the PCA results for all 144 QC samples with color denoting each of the 12 chips used in the six bioprocessor plates. Again there is no apparent systematic variance. Thus, no systematic variability across plates, across chips or the spot positions is discernable from PCA analysis.

The samples of interest in our studies (i.e., the study of biomarkers for liver cancer and liver toxicity, as well as myeloma-associated lytic bone disease) were loaded on the spots of the chips in the bioprocessor plates together with the QC samples. As the analysis of the QC samples showed no systematic variability across spot locations, chips and plates, these sources of variability should have minimum affect on the data analysis for the identification of potential biomarkers that differentiate liver cancer patients from the healthy individuals and patients with bone lesions from those without bone lesions. Thus, the biomarkers and the associated patterns in our study were identified from the protein profiles with little systematic variability (results not shown).

### Assessment of reproducibility of experiments

**Figure 2:**
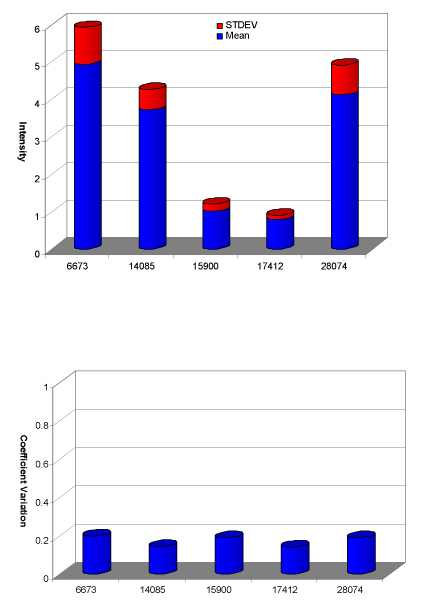
**Demonstration of reproducibility of the SELDI experiments in liver cancer and liver toxicity study. **The reproducibility of SELDI experiments was measured by the consistency of expression levels (peak intensities in spectra) of the five common proteins appearing in all 144 quality control samples used in the liver cancer and liver toxicity study. The numbers on the x-axes are the m/z values of peaks (in KDa.). (**A**) The average intensities of the five peaks in the 144 spectra are represented by the blue cylinders and the corresponding standard deviations depicting the variations in the 144 spectra are shown in red cylinders. (**B**) The coefficient of variation (CV), which is used to measure the precision of the peak intensity common in the 144 spectra from the set of SELDI experiments, is depicted as blue cylinders. All CVs are less than 0.2, which is considered acceptable reproducibility in SELDI.

Identification of systematic variability that introduces noise by affecting the quality of peaks is but one aspect of quality assessment of experiments. Minimal systematic variability is a necessary but insufficient prerequisite to conclude that the quality of the experiment is high and acceptable. Another crucial aspect of quality assessment for experiments is the reproducibility: the measurement of how consistent the results are from the same or similar samples. Biological and technical replicates were used in the SELDI experiments of liver cancer and liver toxicity, as well as myeloma-associated lytic bone disease studies for the purpose of quality control and quality assessment. The reproducibility of 144 spectra from the QC samples assayed on six bioprocessor plates with 12 protein chips each is shown in Fig. 2. Fig. 2A gives the average intensity and corresponding standard deviation of the five m/z peaks detected in all 144 spectra. The intensities of common m/z peaks show small variations. Fig. 2B gives the coefficient of variation of the five peaks, which are all less than 20%, indicating that the SELDI experiments are consistent based on the levels of common proteins expressed in the 144 QC samples. The analysis of the consistency of the experiments demonstrated that high reproducibility was obtained in our SELDI studies.

### Detection of low quality spectra

The reproducibility of experiments measures the overall consistency among a set of spectra obtained from SELDI experiments. However, it does not give any detail on the quality of individual spectra in the dataset. In other words, reproducibility is a good assessment of the overall consistency of a set of experiments, but it can not be used to identify low quality spectra. Low quality spectra need to be removed from the data set prior to analysis in order to reliably discover biomarker proteins and the associated expression patterns that differentiate a disease from control or that distinguish different stages of a disease such as cancer. For assessment of reproducibility, all common peaks have to be (and were) used. But for quality assessment, whole spectra (all possible peaks) must to be used. A Pearson correlation matrix was used for QC of the individual spectra of biological and technical replicates prior to SELDI data analysis. The rationale behind the approach is to assume that the protein expression profiles of biological and technical replicates of a sample are similar and thus that the correlation among spectra of same or similar samples should be very high.

**Figure 3:**
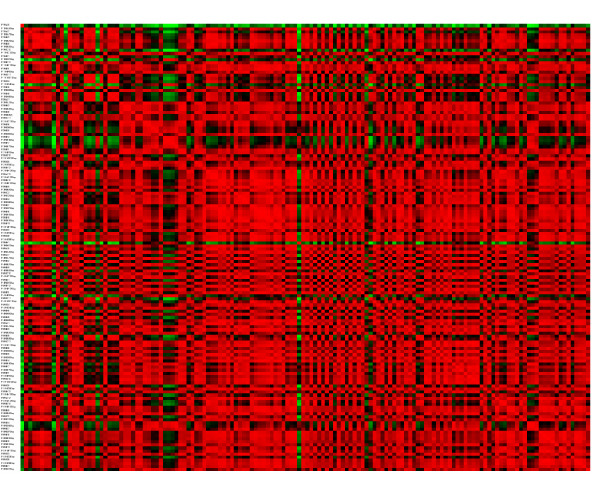
**Heat map of correlation matrix of the 144 spectra from quality control samples. **The Pearson product-moment correlation coefficient (R) is a measure of the degree of linear relationship between two spectra. A higher correlation indicates a more similar protein expression pattern between two samples for the two spectra. Each row and column in the heat map of the correlation matrix represents a spectrum. Each point is a Pearson correlation coefficient, R, between the spectrum in the column and the spectrum in the row. The correlation coefficients were colored by the values of R. The higher the value of R, the brighter red is the data point. The lower the value of R, the brighter green is the data point. All 144 data points in one row (same for column) represent the correlation of one spectrum to all other spectra. Therefore, the green line indicates the spectrum does not correlate well to other spectra. The correlation matrix was calculated using software JMP 5.1  and the heat map was created using the software DMVS 2.0 (Shenzhen Chipscreen Biosciences, Ltd, Shenzhen, P. R. China, )

In our liver cancer and liver toxicity studies, the biological replicates of QC samples were used for both quality control and quality assessment. The correlation matrix of 144 spectra obtained from SELDI experiments of QC samples based on the five m/z peaks common to all 144 spectra is shown as a heat map in Fig. 3. The color red in Fig. 3 denotes high correlation (r > 0.9) between two spectrums, whereas green denotes low correlation (r < 0.9), and black denotes r = near 0.9. The preponderance of red in Fig. 3 indicates that most of the 144 spectra are well correlated with a few exceptions that are shown in green. In fact, the median correlation coefficient was 0.96, with a maximum of 1 and a minimum of -0.14. The standard deviation within the correlation matrix was 0.13.

**Figure 4:**
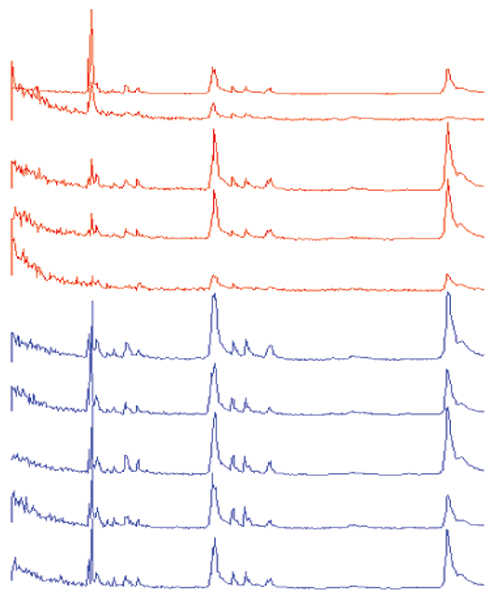
**Representative examples of SELDI spectra from quality control samples. **Examples of spectra illustrate differences between highly consistent spectra and low quality spectra. The spectra in blue are examples of the highly consistent spectra, which include the majority of the 144 quality control samples. All spectra have very similar peak patterns as could be expected for the biological replicates of quality control samples. This indicates that the experiments were conducted consistently and that the spectra are of high quality. In contrast, the spectra in red are the low quality spectra detected by the correlation matrix and that need to be discarded prior to data analysis. All of the low quality spectra are different from the high quality spectra in terms of the peak patterns. Furthermore, unique peak patterns do not exist among the low quality spectra.

**Figure 5:**
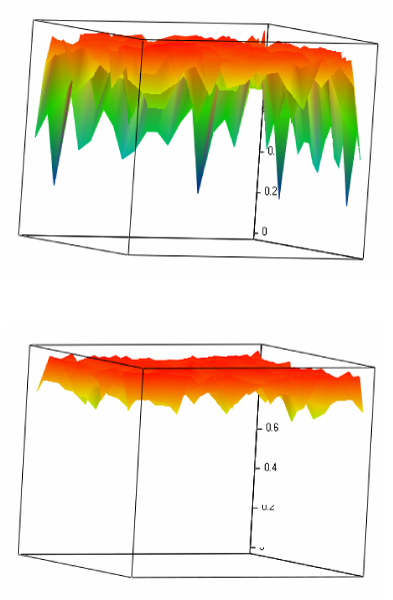
**Comparison of spectral quality before and after low quality spectra are detected by the correlation matrix and discarded. **The quality of spectra from biological replicates of quality control samples is illustrated in the Pearson correlation matrix surfaces that were generated using the software DMVS 2.0 (Shenzhen Chipscreen Biosciences, Ltd, Shenzhen, P. R. China, ). The x and y axes represent spectra and the z-axes indicate the values of Pearson correlation coefficients. The bright red color indicates very good correlation with values of R close to 1, and the green color indicates that a low correlation was observed between two spectra. (**A**) Correlation matrix was applied to the dataset prior to quality control. Green valleys caused by several low quality spectra that had very low correlations to most of other spectra were detected. (**B**) After the low quality spectra were detected by the correlation matrix and discarded, the Pearson correlation matrix surface became clear and very red, indicating that the majority of the correlations among all spectra are very high.

There are several low correlations in the matrix that were expected to be high since all the spectra were obtained from biological replicates of QC samples. These QC samples should have similar protein expression patterns and therefore should be highly correlated SELDI spectra. Low correlations are likely associated with low quality spectra that may be caused in one or more steps in the complicated SELDI experiment and must be discarded prior to further data analysis. To confirm that the spectra with low scores in the correlation matrix are true low quality spectra, which are different from the majority of the 144 QC samples assayed by SELDI, the raw spectra were visually inspected. We found that the spectra detected as low quality spectra in the correlation matrix are indeed different from the others, though the number of such spectra is very small. Fig. 4 compares the low quality spectra (red) with high quality spectra (blue). It can be seen clearly that all high quality spectra are very similar to each other based on the m/z peak patterns, but that the low quality spectra have different m/z peak patterns compared with the high quality spectra. The protein expression pattern observed in the low quality spectra is distinct from the rest of the samples although they should have similar protein expression patterns. From the point of view of data analysis those low quality spectra need to be removed prior to the data analysis for identification of biomarkers. The comparison of data sets before and after the low quality spectra were discarded is shown as spectral correlation matrix surface in Fig. 5. After the low quality spectra were discarded (Fig. 5B) the correlation matrix surface is much smoother, cleaner and bright in red compared to the surface before those spectra were discarded (Fig. 5A).

**Figure 6:**
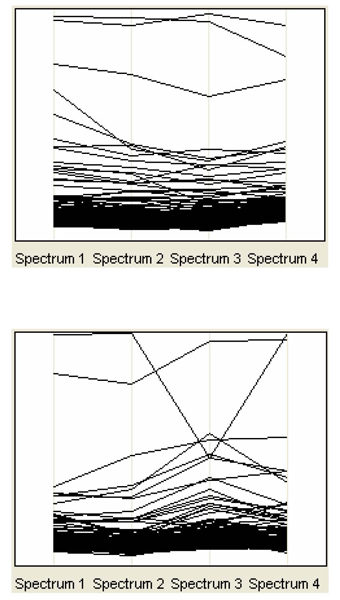
**Examples of high and low quality spectra from technical replicates in myeloma-associated lytic bone disease study. **The parallel coordinate plots of spectra of four replicates from sera of patients X and Y illustrate the difference between the high quality spectra and low quality spectra. Each peak in the spectra of four replicates were drawn as four points and linked together by a line to show the relative trend. The y-axis is the intensity. (**A**) Four spectra from replicates of serum of patient X showed very similar profiles and have a high degree of correlation to each other. (**B**) Spectra 1, 2, and 4 from patient Y have similar profiles and therefore have high correlations to each other. But spectrum 3 has a different profile from the others. Since it is not consistent with the other replicates, it should be discarded prior to data analysis.

In our study of identification of biomarkers for myeloma-associated lytic bone disease, technical replicates were used for quality control purpose to make the identification of biomarkers and associated patterns reliable for potential application in clinical diagnosis. Each serum sample from a newly diagnosed myeloma patient was placed on four adjacent spots of a chip for SELDI assays. The four SELDI mass spectra should be very similar since they are the replicates of the same sample. It was expected that use of technical replicates could serve a QC purpose to ensure all the spectra used in the data analysis were of high quality. Integration of quality control prior to the data analysis improves the quality of the predictive model derived from the data set with respect to prediction accuracy, sensitivity, and specificity (result not reported here). Here we show examples of the application of the correlation matrix as a quality control tool for the technical replicates used in our myeloma-associated lytic bone disease study. To explain how to detect and discard low quality spectrum, spectra from two patients X and Y, one with a low quality spectrum and the other with all high quality spectra were elected as examples. Table 1 lists the correlation matrix of the four SELDI mass spectra obtained from patient X, where high correlations among the four spectra are observed with r greater than 0.97. This indicates consistency between the four SELDI experiments such that high quality spectra for biomarker identification should be expected. In contrast, the correlation matrix indicated low quality interspectrum correlation for patient Y (Table 2), where spectrum 3 has r < 0.9 compared with the other three spectra. However, the other three spectra are well correlated with r > 0.97. The ostensible single low quality spectrum from patient Y suggests that experimental steps were inconsistent, causing its corresponding spectrum to poorly correlate. Accordingly, removal of spectrum 3 should result in more reliable biomarker identification. Fig. 6A and Fig. 6B show plots of peak intensities for patients X and Y, respectively. The similar and consistent spectra for patient X are clearly evident, as are the disparate spectra for patient Y. The correlation matrix is thus an effective metric for identifying the lower quality spectra.

## Discussion

Recently, the scientific community has been using proteomics to improve diagnostic/prognostic capability and to study the underlying biological processes of diseases and toxicology. Because of the complicated nature of the SELDI-TOF MS ProteinChip analysis, stringent and reliable quality assessment (QA) and quality control (QC) methods and procedures undertaken prior to data analysis are critical. The QA/QC analysis should assure that the data has acceptable experimental variability and that incongruent data that are not the result of biology are screened out. Such assurance of data quality is a necessary condition for identification of reliable biomarker expression patterns with clinical utility for diagnosis and prognosis and for toxicity and safety assessment.

Two phases of quality assessment and quality control can be applied in the workflow of SELDI-TOF MS ProteinChip proteomic analysis. The first QA/QC phase is aimed at examining and improving the experimental techniques to produce quality data. This phase incorporates biological and technical replicates to identify and quantify the systematic bias and noise in the experiments; the process spans all experimental steps such as calibration of instruments, proper sample preparation, and so forth. The emphasis of this phase is on improving the quality of the experiments.

The second QA/QC phase emphasises data analysis. Even if stringent procedures are applied in the first phase, it is difficult to guarantee that all spectra from the experiments are of sufficient quality for subsequent data analysis. A thorough quality assessment and quality control process is required in order to ensure that only high quality data are used. Low quality spectra need to be identified and excluded.

A correlation matrix was found to be an effective means to detect low quality spectra among the biological and technical replicates. Use of correlation matrix approach is based on the assumption that spectra of biological and technical replicates should be similar and well correlated, and thus outlying spectra are of relatively lower quality and significance. Our results suggest that inter-spectral correlation coefficient values of R > .95 to .97 are attainable and representative figure-of-merit for quality data. Spectra that are inconsistent with other replicates are likely to have been compromised in one or more of the many complex steps in SELDI, or by such factors as lack of uniformity of the matrix material. While the correlation matrix can identify likely anomalies and noisy spectra for exclusion, it cannot indicate the source of the variability. Even several phase one QC/QA methods such as using pooled QC samples and using standard calibrant to calibrate the spectra were adopted in our studies to make the experiments high quality, the correlation matrix still identified spectra should be excluded from analysis because of low correlation with the other replicate spectra.

The liver cancer and liver toxicity study used 144 QC samples dispersed evenly across six Ciphergen bioprocessor plates and different chips, and randomly located on different spots. The experiments were conducted on different dates. No date effect was observed. The samples were collected into heparinized tubes and stored at -80°C. The period of storage for samples used in this study ranged from several weeks to one year. The experiments and the quality assessment analysis showed that the samples were very stable during sustained storage.

## Conclusion

Application of new technologies such as SELDI-TOF MS coupled with the ProteinChip for biomarker identification requires a robust QA/QC assessment process. QA/QC pre-processing is critical for discovery of biomarkers and expression patterns that have potential use in clinical settings, as well as in toxicity studies for safety assessment. In this report, we described a method for monitoring the quality of proteomics profiles from SELDI-TOF MS. The results showed that the reproducibility of our SELDI experiments studies of liver cancer and liver toxicity was high with coefficients of variation less than 20% for five common proteins across all 144 QC samples. In addition, we found no systematic variability across plates or chips, or spot locations. The quality assessment lent confidence that the data obtained met requirements for biomarker identification. To ensure that all data used in the data analysis for the discovery of biomarkers are of high quality with low noise, the low quality spectra should be identified and removed prior to data analysis. We developed a method using a correlation matrix to identify the low quality spectra observed in biological and technical replicates. The correlation matrix approach was applied to the liver cancer and toxicity study and the myeloma-associated lytic bone disease study to efficiently identify low quality spectra prior to post-analysis.

## Authors' contributions

HH had the original idea on the methodology, did all calculations and analysis, and wrote the first draft of manuscript. YD had the original idea for the liver cancer and liver toxicity study, and designed and coordinated the experiments. CT did the experiments for the liver cancer and liver toxicity studies. JE had the original idea for the myeloma-associated lytic bone disease study, and designed and coordinated the experiments. BC carried out the experiments for myeloma-associated lytic bone disease study. WT, QX, HF, LS, and RP were involved in and contributed to the approach used in the data analysis. WT, RP, YD, JE, and BC assisted with writing the manuscript. All authors read and approved the final manuscript.

## Figures and Tables

**Table 1:** Pearson correlation matrix of four spectra from patient X. Column "Mean" lists the average Pearson correlation coefficients of each spectrum to other spectra.

	Spectrum 1	Spectrum 2	Spectrum 3	Spectrum 4	Mean
Spectrum 1	1	0.981669	0.966975	0.976304	0.981237
Spectrum 2	0.981669	1	0.982505	0.984483	0.987164
Spectrum 3	0.966975	0.982505	1	0.984252	0.983433
Spectrum 4	0.976304	0.984483	0.984252	1	0.98626

**Table 2:** Pearson correlation matrix of four spectra from patient Y. Column "Mean" lists the average Pearson correlation coefficients of each spectrum to other spectra.

	Spectrum 1	Spectrum 2	Spectrum 3	Spectrum 4	Mean
Spectrum 1	1	0.974029	0.832734	0.973365	0.945032
Spectrum 2	0.974029	1	0.857734	0.979373	0.952784
Spectrum 3	0.832734	0.857734	1	0.897082	0.896888
Spectrum 4	0.973365	0.979373	0.897082	1	0.962455
